# Supplementary material for: Apoptotic Effect of Combinations of T-2, HT-2, and Diacetoxyscirpenol on Human Jurkat T Cells
Source: Toxins (Basel). 2025 Apr 18;17(4):203. doi: 10.3390/toxins17040203 (PMC12030997; doi:10.3390/toxins17040203)
Supplement: Supplementary file 1 [file toxins-17-00203-s001.zip › toxins-3535665-supplementary.pdf]

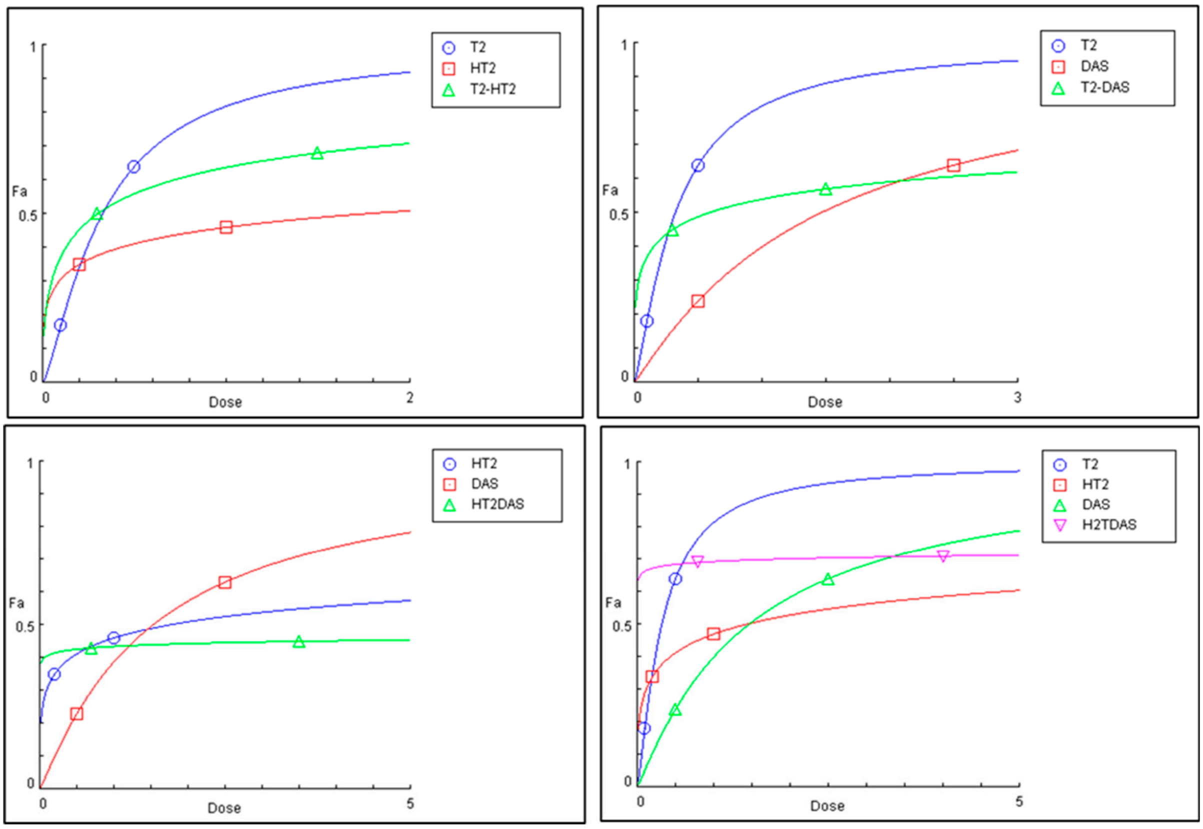

Figure S1. Dose – effect relationship for T-2, HT-2 and DAS and their mixtures in relationship to cell viability of human Jurkat T cells.
